# Modeling Evolutionary Dynamics of Epigenetic Mutations in Hierarchically Organized Tumors

**DOI:** 10.1371/journal.pcbi.1001132

**Published:** 2011-05-05

**Authors:** Andrea Sottoriva, Louis Vermeulen, Simon Tavaré

**Affiliations:** 1 Computational Biology Group, University of Cambridge, CRUK Cambridge Research Institute, Li Ka Shing Centre, Cambridge, United Kingdom; 2 Laboratory for Experimental Oncology and Radiobiology (LEXOR), Center for Experimental Molecular Medicine (CEMM), Academical Medical Center (AMC), Amsterdam, The Netherlands; University of California San Diego, United States of America

## Abstract

The cancer stem cell (CSC) concept is a highly debated topic in cancer research. While experimental evidence in favor of the cancer stem cell theory is apparently abundant, the results are often criticized as being difficult to interpret. An important reason for this is that most experimental data that support this model rely on transplantation studies. In this study we use a novel cellular Potts model to elucidate the dynamics of established malignancies that are driven by a small subset of CSCs. Our results demonstrate that epigenetic mutations that occur during mitosis display highly altered dynamics in CSC-driven malignancies compared to a classical, non-hierarchical model of growth. In particular, the heterogeneity observed in CSC-driven tumors is considerably higher. We speculate that this feature could be used in combination with epigenetic (methylation) sequencing studies of human malignancies to prove or refute the CSC hypothesis in established tumors without the need for transplantation. Moreover our tumor growth simulations indicate that CSC-driven tumors display evolutionary features that can be considered beneficial during tumor progression. Besides an increased heterogeneity they also exhibit properties that allow the escape of clones from local fitness peaks. This leads to more aggressive phenotypes in the long run and makes the neoplasm more adaptable to stringent selective forces such as cancer treatment. Indeed when therapy is applied the clone landscape of the regrown tumor is more aggressive with respect to the primary tumor, whereas the classical model demonstrated similar patterns before and after therapy. Understanding these often counter-intuitive fundamental properties of (non-)hierarchically organized malignancies is a crucial step in validating the CSC concept as well as providing insight into the therapeutical consequences of this model.

## Author Summary

Cancer is in essence a genetic disease that leads to uncontrolled cell proliferation, invasion and metastasis. The cancer stem cell (CSC) hypothesis states that tumors are not just a mass of uniform malignant cells but they are hierarchically organized, like normal tissues. At the top of such a hierarchy are cancer stem cells that fuel tumor growth in the long run, whereas the majority of other cells are able to divide only a few times. The experiments that support the CSC hypothesis are often criticized as being difficult to interpret. A novel approach to test the CSC paradigm is to integrate mathematical modeling with DNA variation data that carry the phylogenetic history of cells. We have developed a model that simulates the occurrence of such changes under both the CSC hypothesis and the classical, purely stochastic scenario. We found that although a CSC-driven tumor has a smaller number of tumorigenic cells, it triggers more malignant properties such as invasive growth, heterogeneity and evolutionary escape from peaks in the fitness landscape. These properties, that are unique to the CSC model, are enhanced even further when a treatment is applied to the tumor.

## Introduction

Tumor formation and progression are highly dynamic processes that are driven by the accumulation of genetic lesions that facilitate the ability of cancer cells to invade surrounding tissue, form metastases and develop resistance to therapy. As suggested by Nowell [1], cancer progression is driven by selective pressure on the cancer cell population as a result of the competition for space and resources among different malignant cells as well as normal cells. Recent observations complicate this attractive model by suggesting that besides clonal (genetic) variations between cancer cells, the differentiation grade of cells also contributes to the heterogeneity found in tumors of various kinds [2], [3].

Experiments demonstrating that only a small fraction of cancer cells are capable of transplanting the disease in immuno-compromised mice led to the speculation that tumors are hierarchically organized tissues that depend on so-called cancer stem cells (CSCs) for their long-term growth. This assumption is supported by the finding that tumor initiating cell populations can be isolated based on expression of markers that are often associated with immature cell types in a variety of tissues. For example only the CD133^+^ fraction of glioblastoma cells, which make up approximately 1% of the total tumor cell load, is capable of initiating growth of a new glioblastoma upon transplantation. Injection of as many as 100,000 CD133^+^ cells does not result in effective tumor formation [4]. To date, the evidence for the CSC model of malignancies is entirely based on transplantation assays [5], [6].

Problems with interpretation of the transplantation data include potential xenotransplantation bias (injection of human cells into mice), remaining immunological effects in the recipient mice, and the fact that for isolation of the various cell populations all the tumor tissue is disrupted. Moreover the fact that the main tumor mass is not capable of initiating a new tumor does not necessarily imply that these cells are also incapable of participating in the growth of an established malignancy. Indeed it is found that various mouse models of hematological malignancies do not display a rare CSC compartment when the cells are injected in autologous mice [7]. Moreover it appears that the type of immuno-compromised mouse strain used for the transplantation assay greatly influences the fraction of cells capable of inducing tumor growth [8]. This has led numerous researchers to warn against overly optimistic interpretations of these data, and has resulted in intense debate in the oncology field over the validity of the CSC concept [5], [9], [10], [11].

Previously, we and others have demonstrated how the analysis of methylation patterns in regions of the genome rich in CpG dinucleotides (a molecular pattern that can be methylated), collected from different parts of a tumor, can be a valuable tool in deciphering the phylogenetic history of malignancies [12], [13], [14]. The advantage of methylation is the higher mutation rate of ∼2×10^−5^ per site per division [15] compared to DNA point mutations, such as microsatellites (∼10^−10^ [16]). This makes methylation changes occurring in neutral CpG-rich genomic regions a powerful and more precise molecular clock that acts as a cell division counter and allows for the inference of cell phylogeny within established malignancies. The neutrality of these mutations, i.e. the fact that these loci are replicated but not transcribed and expressed, is important because selective forces may disrupt the relationship between the number of errors and of cell divisions. Being suitable markers to trace cell fate in tumors, in this study we model the occurrence of neutral methylation mutations in respect of pattern heterogeneity and distribution with the aim of comparing the dynamics of a CSC-driven malignancy to a purely stochastic, non-hierarchical model of tumor growth that we refer to as the *classical model*.

In addition to concerns related to the experimental procedures that support the CSC theory there are also important theoretical arguments that challenge the CSC concept. Classical organized clones, in which all cells are clonogenic, are predicted quickly to outgrow the hierarchical clones due to favorable growth kinetics. Therefore, one would expect that evolutionary forces rapidly select for non-hierarchical cancer cell populations that outcompete the stem cell driven clones that have retained this organization from the tissue in which they arose. However, potentially CSC-driven tumor cell clones display non-intuitive features that are useful in the process of tumor formation and perhaps even in therapy resistance. To investigate the different evolutionary dynamics of the two models and to explore the potential evolutionary benefits of a hierarchical organization of cancer cells, we expand the computational model and introduce non-neutral mutations that can confer a different fitness on cells. In this setting we analyze the behavior of the different models under different fitness conditions and landscapes. To conclude, we also study the effect of therapy on CSC-driven tumors.

## Results

We developed a model of tumor growth based on a cellular Potts approach that can simulate cancer cell proliferation in a realistic fashion. Our model incorporates the fundamental processes occurring in cancer growth, such as cell division and apoptosis. Cellular Potts models have the important benefit of being able to simulate complex cellular mechanisms such as cell division, apoptosis and cellular rearrangements realistically and very efficiently, without the need of artificial assumptions on cellular mechanics. On top of this we model the occurrence of neutral methylation mutations and the existence of a hierarchical organization in the malignant clone, composed of cancer stem cells (CSC), transient amplifying cells (TAC) and terminally differentiated cells (DC) (see Materials and Methods).

To study the dynamics of neutral epigenetic mutations as markers of cell populations in cancer [17] we model neutral methylation changes by assuming that at each cell division errors in copying a 64-CpG dinucleotide region can occur in both the mother and the daughter cell. We assume methylation and demethylation rates of µ = 2×10^−5^ errors per CpG site per cell division occurring in all cells, regardless of their proliferative potential or their stemness [15]. To simulate hierarchical tumor organization we assume that CSCs in the system are able to self-renew with probability ψ or to divide asymmetrically with probability 1-ψ. In the first case the result of the cell division are two CSCs, in the second the original CSC and a TAC, able to divide only *G* times before becoming quiescent. Due to the absence of homeostasis in the tumor we assume that symmetrical division yielding two transient amplifying cells (CSC differentiation) does not occur. With this scheme classical clonal tumor growth in which all cells are tumorigenic is simulated by setting ψ = 1 (see Materials and Methods for details). The model parameters are summarized in Table 1.

**Table 1:** Parameters of the cellular Potts model.

Parameter	Symbol	Value	Reference/Justification
Cell cycle duration	*c*	20 h	[52]
Methylation rate (per CpG per cell division)	*µ*	2×10^−5^	[15]
Cell adhesion coefficient	*J*	9	[53]
Cell stiffness	*λ*	3	Derived from [53]
Maximum number of cell divisions per TAC	*G*	5 (3, 7 in **Figure S3C**)	Vary *G* and ψ to vary CSC fractions [2], [18]
Apoptosis rate (fraction per 24 h)	*a*	0.01 (0, 0.04 in **Figure S3A-B**)	Rate lower than CSC growth rate
Apoptosis rate (fitness)	*a_f_*	0.02	Rate lower than CSC growth rate
Non-neutral mutation rate	*µ_f_*	0.1	[28]

### Neutral epigenetic mutations

**Figure 1:**
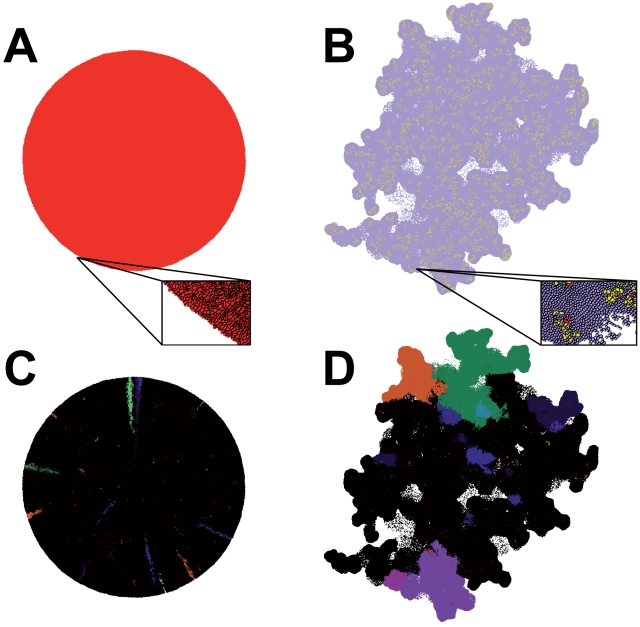
Morphology of the classical model and the CSC model. Tumor morphology appears spherical in the classical model (**A**) whereas tumor borders in the CSC model are irregular (**B**). Red: CSCs, yellow: TACs, blue: DCCs (zoom box, black: cell borders). The distribution of the neutral methylation patterns is radial in the classical model (**C**) versus patch-like in the CSC one (**D**).

We used our cellular Potts model to investigate levels and distribution patterns of epigenetically distinct clones in both CSC-driven malignancies as well as in non-hierarchical organized tumors. Figure 1A and Video S1 display the simulated growth of a classical malignancy with ψ = 1 whereas Figure 1B and Video S2 shows a CSC malignancy with ψ = 0.1. The total tumor volume in both experiments is 100,000 cells. While the classical model exhibits a spherical morphology, a CSC-driven neoplasm is characterized by irregular tumor borders and invasive patterns driven by expansion of CSCs, as already reported in previous studies by us [18] and others [19]. With respect to the methylation patterns the two models also behave in a radically different way. The CSC model shows a patch-like distribution that originates from single founder CSCs while the clones in the classical model tend to follow a radial expansion pattern, as can be appreciated from Figure 1C (Video S3) and 1D (Video S4) in which epigenetically distinct clones are indicated by different colors. The CSC model is also characterized by slower tumor growth and less cell divisions per unit time due to the smaller population of long-term dividing cells (Figure S1A and S1B). The difference in population size between the two models can be summarized by the CSC fraction present in the clone defined as the ratio between the number of CSCs and the total volume at each point in time. In the classical model this value is always 1 whereas in the CSC model it can be variable; in our analysis it quickly stabilizes to ∼0.2% for ψ = 0.1 and *G* = 5, this value is in line with experimental estimates of CSC fractions (Figure S1C) [2], [3].

**Figure 2:**
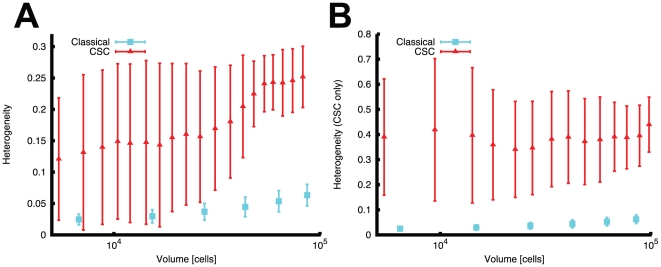
The CSC model enhances methylation pattern heterogeneity. Despite its much smaller effective population size, the CSC model (red) shows consistently higher heterogeneity (**A**) with respect to the classical (blue) model of malignancies (p = 10^−7^ at 100,000 cells, a = 0.01). Importantly this measure is even enhanced when considering the CSC compartment only (**B**) (p = 10^−7^ at 100,000 cells). Error bars represent SD with *n = 16*.

Next we determined the overall heterogeneity of the two different tumor growth models by calculating the Shannon index. The Shannon index [20] is the most frequently applied measure of heterogeneity in biodiversity studies used to describe a population consisting of individuals of genotypically different subpopulations. This measure is based on information theory and possesses properties that account for species richness within an environment. In our case we measure the Shannon index of the cancer cell population and normalize it to the interval [0,1] where 0 indicates a homogeneous population with only one clone and 1 a fully heterogeneous population where all the subclones are equally present (see Materials and Methods). This measure indicates that the CSC malignancy develops a much higher epigenetic heterogeneity both in the total population (Figure 2A) and in the CSC compartment alone (Figure 2B), compared to the classical model (n = 16, p = 10^−7^ for 100,000 cells). The CSC model reaches levels of heterogeneity that are 25% of the maximum possible heterogeneity. These differences can also be appreciated when the non-normalized Shannon index is considered (Figure S2A and S2B). The high standard deviations reported in these plots also suggest that a high level of stochasticity is exhibited by growth driven by a small number of CSCs. As we will discuss further, this feature of CSC driven tumor growth has critical consequences in the evolutionary dynamics of malignancies when non-neutral mutations are considered.

The enhanced heterogeneity is especially striking since within the CSC model the effective population size, i.e. the number of cells contributing to tumor growth in the long run (the CSCs), is about 500 times smaller than in the non-hierarchical model. Moreover we assumed the mutation rate for an individual cell is equal in both models. We confirmed that these results are not dependent on the particular set of parameters we applied, as we observe a similar outcome for different values of the number, *G*, of transient amplifying stages (Figure S3A). Moreover, different apoptosis rates for both the classical and the CSC model, or varying the mutation rate does not change our overall conclusions (Figure S3B, S3C and S4). Interestingly, the parameter *G* has a non-trivial effect on the hierarchical model: high values of *G* correspond to long-living TACs that add proliferative potential to the clone. On the other hand small values of *G* may also increase the CSC ratio due to the small number of cells produced by TACs. In addition, a higher apoptosis rate induces higher heterogeneity by stimulating more cell divisions in both models; however difference was significant only for the CSC model (p = 0.01).

We propose that the increased heterogeneity in the CSC model is due to the fundamental intrinsic property of hierarchical growth models that are driven by long-lived CSCs that must undergo a large number of cell divisions to keep fueling the growing cancer population and thereby acquire more (epi)genetic hits. On top of this mechanism, the probability that a specific clone takes over a subregion of the tumor of size *N* purely by drift, i.e. in a scenario of neutral mutations, is ∼*1/N* whereas for the CSC model is ∼*1/(Nψ)* due to the limited proliferative potential of TACs. Hence, under equal environmental conditions and mutation rate, a CSC-driven tumor can achieve higher epigenetic heterogeneity solely due to its hierarchical organization. This is despite the smaller effective population size of a CSC-driven malignancy. This feature, as well as the distribution of methylation patterns (Figure 1C and 1D) could potentially be used as a signature of a CSC-driven malignancy in established human tumors.

### Non-neutral mutations over a fitness landscape

So far we have shown how hierarchical organization of malignant cells has a major effect on the heterogeneity and spatial distribution of neutral methylation patterns. To study the difference between the two models in term of evolutionary dynamics, we now consider non-neutral epigenetic mutations that confer changes in terms of cell fitness.

Because of the complex interaction between genetic loci, mutations can be mutually deleterious yet confer a fitness advantage when they occur together [21]. Other mutations appear to be mutually exclusive, suggesting that co-occurrence of these genetic alterations confers a fitness disadvantage [22]. The fitness landscape is defined as a map between the space of possible mutations and the fitness advantage conferred by the phenotypes to which they relate. The fitness landscape involved in initiation and progression of malignancies is believed to be a complex curve, with valleys, peaks and local minima and maxima [21], [23]. To represent the effects of different fitness landscapes on a growing cancer we assume that as the population of cancer cells introduces new mutations, the fitness of individuals moves across a certain fitness landscape function *f(x)*. We approximate the evolutionary process by assuming that changes in fitness can occur only by mutations that cause local movements within the fitness function, such as *x* → *x+1* or *x* → *x−1*.

We summarize the complex mechanisms behind the accumulation of epigenetic mutations into a non-neutral mutation rate parameter µ_f_ = 0.1 per cell division that induces changes in the cell phenotype *x*, that in turn corresponds to a division rate *f(x)*. In this scenario clones with different replication times compete with each other for space. A further selective force is represented in our model by apoptosis, occurring at a constant rate that affects relatively slowly dividing clones more dramatically. To illustrate this, we assume a space of 50 phenotypes with *x* ∈ [−24,+25] with solid boundary conditions (no mutation can occur beyond the borders) on which we define different fitness landscapes, both linear and non-linear as well as symmetric and asymmetric, and we compare the behavior of the two models of growth under such fitness conditions. We start all the simulations with a single cell possessing the phenotype *x_0_ = 0* that can randomly move right or left along the x-axis. We simulate growth of the neoplasm until a volume of 100,000 cells is reached (see Table 1 for the other parameters used).

**Figure 3:**
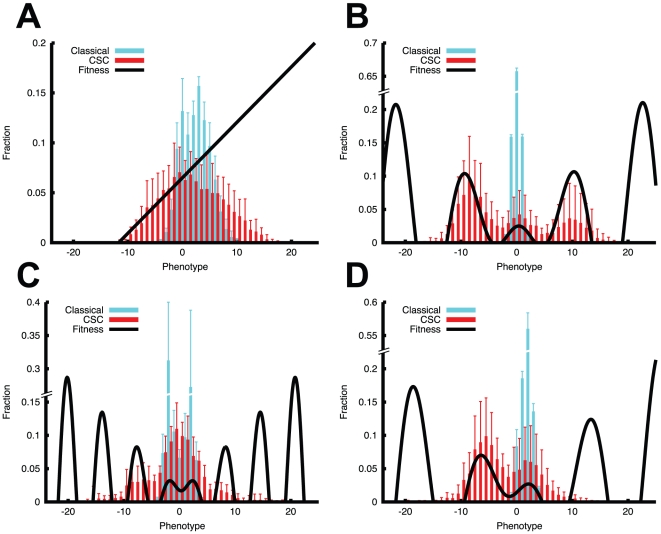
The CSC model escapes local fitness peaks and achieves better fitness in the long run. Within a linear fitness function *f_L_(x) = x+8* (**A**) the CSC model tends to spread towards low fitness regions too, rather than just selecting for the fastest replicating clone. In the case of a symmetrical fitness function with peaks and valleys *f_S_(x) = 3−x sin(x/2)* and *f'_S_(x) = x sin(x)+2* (**B,C**) the CSC model shows evolutionary superiority and the ability to escape local peaks and reach higher fitness in the long run. Even more clearly, the same evolutionary differences are present under an asymmetrical fitness function *f_C_(x) = x cos(x/2)+2* (**D**). Error bars represent SD with *n = 8*.

In the simplest case we define a linear fitness curve *f_L_(x) = x+8* (with *f_L_(x) = 1* for negative values of *x*) in which the replication rate increases proportionally to *x* (Figure 3A, *n = 8*). As expected, in both models the bulk of the population tends towards clones expressing higher fitness (high values of *x*). Importantly, it is apparent how the CSC model shows the property of spreading much faster across the fitness landscape compared to the classical model. The CSC model mainly explores higher fitness regions, yet also phenotypes with relatively low fitness values are abundantly present. This is in line with our previous finding that the CSC model stimulates heterogeneity and we argue that this effect has to do with the lower selective pressure present in the CSC model that allows clones with no direct survival benefit to coexist in the neoplasm and contribute to tumor growth. Furthermore, this peculiar property has consequences in the evolutionary process because it allows populations to visit, and eventually cross, regions of the landscape with lower fitness values that nevertheless may lead to beneficial phenotypes in the long run (fitness valleys).

To evaluate the response of the models to more complex fitness variations we defined a sinusoidal function *f_S_(x) = 3−x sin(x/2)* (with *f_S_(x) = 1* for negative values). This function is characterized by fitness valleys that lead to peaks with faster replicating phenotypes in the long run. An example of a fitness valley is the cooperation of the oncogenes Myc and RAS in mice [24]. Whereas these oncogenes promote tumorigenesis when both are mutated, the mutation of only one of them has an anti-cancer effect due to the induction of apoptosis in Myc-mutated cells and senescence in RAS-mutated ones. From these types of study it is concluded that the crossing of fitness valleys is therefore an important mechanism that occurs during tumorigenesis and tumor progression. To simulate this property we apply a sinusoidal fitness function (Figure 3B, *n = 8*). Here we observe how the evolutionary properties of the classical model prevent clones from overcoming local minima due to the stringent action of phenotypical selection. The cancer cell population can evolve only within the restricted central part of the fitness landscape that represents the local optimum. Strikingly, because of the weaker effect of selection and the more important role of drift, the CSC population can expand to a broader part of the fitness curve and acquire more aggressive phenotypes in the long term. A very similar pattern is illustrated in Figure 3C where the fitness peaks are more numerous due to a slightly different fitness function *f'_S_(x) = x sin(x)+2*. Here again the classical model adapts to the local fitness landscape whereas the CSC model can escape it and reach further evolutionary peaks. This dynamical evolutionary mechanism, driven not only by selection but also by drift, is even more evident when we analyze different growth stages of the experiments presented in Figure 3C (Figure S5). This highlights how the distribution of the clones dynamically *probes* the fitness landscape and ultimately reaches higher fitness points.

To perturb the symmetrical effects of random mutations we define a third function with asymmetric properties to further analyze the response of the models. The fitness landscape *f_C_(x)  = x cos(x/2)+2* specifies a similar function to the previous one but it has asymmetrical properties with respect to the y-axis (Figure 3D). Once more the results suggest a conservative behavior of the classical model that quickly adapts to the local maximum and, in contrast, a highly dynamical behavior of the CSC model that instead overcomes fitness valleys during a fast exploration of the fitness landscape.

To eliminate the bias introduced by an expanding population we investigate the stationary distribution of the phenotypes in a non-expanding malignancy. For this we run a set of simulations with the sinusoidal fitness function *f_S_(x)* in which we start with a volume of 25,000 cells and maintain constant population size by randomly killing cells to maintain the original volume, until a total of 1.2 million cell divisions is reached in both models of tumor growth. This approach shows that the findings we described for expanding malignancies are confirmed for a stationary scenario with constant population size and an equally large number of cell divisions in both models (Figure S6).

Clearly, in both models the clone distribution does not simply scatter through dispersion but is driven by the fitness function that offers local peaks near the starting point with relatively easy access and other higher peaks that are preceded by low fitness values. While the classical model remains trapped within those initial local maxima, the CSC model is free to move ahead to higher fitness peaks, showing its ability to overcome fitness valleys. In all our results, the classical model emerges as a model of growth primarily driven by selection for the (non-optimal) fittest clones that outcompete the large number of tumorigenic cells in their neighborhood. In this type of scenario overcoming local maxima and exploring large portions of the fitness landscape becomes impossible. Within high selective pressure conditions, any cell that acquires even a slightly lower fitness (approaching a fitness valley) due to a disadvantageous mutation would be immediately out-competed by the surrounding cells with higher fitness. The higher robustness of the CSC model, induced by its lower selective pressure that allows for the proliferation of several clones with no selective advantage, increases the adaptability of the cancer population that also becomes more evolvable. The lower selective pressure allows cells to approach a fitness valley and bear *temporarily* disadvantageous mutations such as RAS with wildtype Myc [24] to survive in a sort of spatial evolutionary niche. Such cells could then carry on accumulating aberrations, such as Myc in this example, and become more malignant in the long run. This direct but counterintuitive relationship between robustness and evolvability has recently been discussed by Draghi and colleagues [25]. In a CSC-driven malignancy such robustness is not assumed *a priori*, depending on a specific fitness landscape, but emerges naturally from the hierarchical organization itself. These results show how a hierarchically organized populations of cancer cells, despite having the disadvantage of a smaller effective cell population size, can escape stringent selective forces represented by cancer therapies that target fast dividing cells. Such treatment modalities would be highly effective in a selection-driven classical model of growth. This therapy-escaping mechanism present in hierarchical malignancies may lead to a more invasive and therapy-resistant cancer through faster accumulation of diverse clones and evolutionary escape of local fitness peaks.

### Evolutionary dynamics associated with treatment

**Figure 4:**
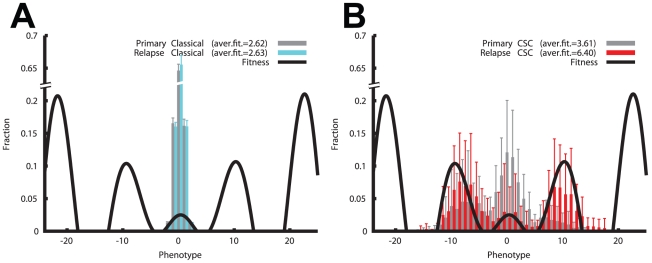
The CSC model stimulates malignant features in relapsing tumors after therapy. Whereas the relapsing tumors in a classical model are highly similar to the primary ones, displaying an unaltered average fitness (**A**, p = 0.04), the CSC model not only shows a different clonal distribution, but also the average fitness is considerably increased (**B**, p = 0.0023). Error bars represent SD with *n = 12*.

To investigate the influence of therapy in the different cancer scenarios we simulate the application of treatment when the tumor volume reaches 30,000 cells. At that point in time, all proliferating cells (see Material and Methods) are killed, independently of their differentiation status. The remaining cells are left to repopulate the tumor and form the relapse. We analyze the status of the tumor just before therapy and after the relapsed neoplasm has again reached the volume of 30,000 cells. As an example we consider the sinusoidal fitness function *f_S_(x)* with the same conditions as employed before. Whereas the classical model displays no evident difference between the two time points (the neoplasm has just regrown similarly to its primary counterpart), in the CSC model the therapy has radically altered the distribution of the phenotypes, pushing larger populations of cells to occupy higher fitness peaks (Figure 4A and 4B). The phenotypical heterogeneity of the clones does not significantly change in the CSC model (p = 0.46) while it decreases slightly in the classical model (p = 0.02) (Figure S7A). However, the average fitness does increase considerably in the CSC model (p = 0.0023) and yet only minimally in the classical model (p = 0.04) (Figure S7B). According to this analysis the CSC model not only relapses more aggressively, but it also changes the phenotypical composition of its clones, resulting in a relapsed neoplasm that is radically different from the originally treated one. In the classical model most of the cells stay within the local fitness peak seeking the highest local fitness point. Cells that eventually survive therapy are still trapped within the same peaks and the tumor that develops after relapse is very similar to the primary one. Said differently, the CSC model also allows less fit clones to survive in the lower parts of the fitness landscape. In this way cells are not only prone to cross fitness valleys, but in case of treatment, they can survive due to their slow cycling phenotype. Hence, the relapsed tumor benefits from the further expansion of the clone pool towards even more aggressive phenotypes. This mechanism results not only in the increase of aggressiveness of the neoplasm at time of relapse, but also in a very different clonal composition of the tumor. The behavior of the CSC model therefore suggests that the fundamental organization of malignant clones may directly influence the therapeutic effect of treatment and the acquisition of resistance in cancers.

## Discussion

Modeling tumor growth using cellular automata, partial differential equations and hybrid models has revealed some of the important underlying dynamics of the growth of malignancies [26]. Since the models proposed by Dormann and Deutsch [27] it has been possible to simulate the formation of a three-layered structure in tumors, made by proliferating, quiescent and necrotic layers. Successively, more advanced hybrid models have shown how the invasion patterns in malignancies could arise from the interaction with the microenvironment [28]. A similar hybrid approach, incorporating the CSC concept, has illustrated how a small population of stem-like cells in cancer can drive tumor invasion with a mechanism that the authors define as self-metastasis [19]. Using a cellular automata approach we have previously shown that tissue invasion and intra-tumor heterogeneity could arise from the internal organization of the clone itself, from its interaction with the microenvironment or from a complex mixture of both [18], [29].

Recently, cellular automata based on Potts models [30] have been developed to simulate the dynamics of populations of cells [31]. Models derived from this paradigm have been successfully employed in cancer research to predict the conditions for cancer cell survival and the diffusion dynamics of growth factors [32]. In this work we use a similar approach to model the cellular growth of malignancies but for the first time to our knowledge, we integrate the cellular Potts modeling approach with the hierarchical organization of cancer cell populations and with the occurrence of neutral and non-neutral mutations to study the evolutionary dynamics of malignancies. We put special emphasis on the process of cancer evolution [33] since this covers the most fundamental questions in cancer research such as the progression of tumors to invasive and metastatic neoplasms.

In evolutionary terms, cancer cells compete for resources such as oxygen, glucose and space, both with other cancer clones as well as with normal cells surrounding the malignancy. The limit of available resources, such as the space to proliferate, induces a selective pressure on the expanding neoplasm. Each cancer cell competes for proliferation within those limits, a mechanism summarized in our model with the space constraint and random apoptosis. In this context, malignant cells with higher fitness can outcompete their neighbors in terms of proliferation and so lead to clone expansion. Hence, a fully clonogenic tumor, i.e. a tumor in which all cells posses the capacity to proliferate indefinitely, appears to be the best organization to develop further malignant traits and clonal heterogeneity, as observed in tumors *in vivo* [34], [35], [36].

However, our investigations suggest that under the same environmental conditions and mutation rate, the evolutionary dynamics of neutral epigenetic changes in tumors lead to the counter-intuitive emergence of intra-tumor heterogeneity in hierarchically organized malignancies. This process seems to be intrinsic to the organization present in the malignant clone itself and need not depend upon external microenvironmental factors. Certainly, the tumor microenvironment has been shown to play an important role in cancer, in particular in modulating stem cell features of cancer cells [37], [38]. Although we do not take this into consideration in the present study, previous results suggest that a model where the stemness is intrinsic to the cell and a model where instead it is completely determined by the microenvironment yield similar results regarding the clonal evolution of the malignancy [29]. In our future studies we plan to incorporate this aspect of tumor biology in more detail; in fact our cellular Potts model approach is a natural framework to study this type of interaction.

The CSC compartment may be fixed or display plasticity driven by the microenvironment; in both cases a tumor organization that stimulates heterogeneity through genetic drift [18], [33] and promotes the emergence of clones without a direct survival benefit may allow a more malignant evolution of a cancer cell population [39]. Furthermore, we have shown how in the CSC model cancer cells are free to explore the landscape of possible genetic alterations, overcome fitness valleys (local minima) and escape from local fitness peaks (Figure 3, S5 and S6), as also observed in the evolution of small populations [40] and in the adaptability of robust phenotypes [25]. We believe that such growth could give rise to radically different evolutionary dynamics, unlike the scenario where only the fittest individuals overcome the competitors. Instead, we propose that populations of cancer cells can raise their survival chances not only through their relative fitness, but also by increasing genetic drift through a mechanism of segregation. In this way, individuals could achieve survival because of the lowered selective pressure and increased genetic drift, rather than through stringent competition. This last mechanism can occur by means of migration but also through hierarchical organization of clones that consequently would occur in segregation of individuals through asymmetric division of stem-like cells.

In a highly competitive environment with scarce resources such as in a tumor microenvironment, being fitter (e.g. proliferating faster) may be too expensive and/or disadvantageous in the long run since it diminishes the heterogeneity of the total population, as we demonstrated in this study. Alternatively, a clone could evolve through an easier yet riskier path, such as due to lower selective pressure and increased genetic drift induced by any mechanism of segregation. This suggests an intimate relationship between evolution and organization of a malignant clone: the hierarchical structure of growth could be advantageous in evolutionary terms with respect to a flat structure, despite its apparently limited proliferative potential. Generally, this process could occur in other types of evolving populations. These findings directly support the need for using spatial models in the study of evolutionary dynamics of tumor growth.

On top of these mechanisms, a hierarchically organized cancer population has the capacity to regenerate after treatment, presenting a relapsed neoplasm that displays more aggressive traits and a substantially different clonal composition compared to its primary counterpart. This is especially intriguing as relapsed tumors are often considerably different from the original malignancy in many features including aggressiveness and clonal composition. This fact is usually attributed to the selection of clones that are relatively resistant to particular drugs during treatment [41], [42], [43]. However, our findings indicate that a CSC-driven malignancy has intrinsic features that explain such behavior independently of clonal resistance.

In conclusion this work presents a theoretical study of the emerging properties of CSC-driven tumor growth. It clearly points out interesting and counterintuitive features of hierarchically organized models of growth. To corroborate our findings by means of experimentation, a possible approach would be to employ extensive methylation sequencing [15], [17] of *in vitro* or *in vivo* tumors known to retain a CSC organization [44]. Our predictions could help to establish if rare CSCs are effectively present in tumors, as advocated by the CSC hypothesis. More importantly, our results refer directly to an *in vivo* context that can be accessed in terms of methylation analysis, a scenario that transplantation and *in vitro* experiments are unable to reproduce convincingly. Finally, our study elucidates evolutionary mechanisms that have critical implications for therapy resistance of tumors such as the increased heterogeneity, the escape from local fitness peaks and the capacity to present a completely altered malignancy after treatment.

## Materials and Methods

### The tumor growth model

To simulate a malignancy and the mutations occurring in it we developed a mathematical model of tumor growth based on a *Cellular Potts Model* (CPM) [30]. A CPM is a Monte Carlo computational modeling technique developed in the field of statistical mechanics. Our model allows us to simulate several important processes occurring in the growth of a tumor such as cell proliferation, cell membrane deformation and cell-to-cell adhesion. A CPM represents the system, in our case the tumour, as a 2-dimensional lattice Ω with N×N sites. Each cell has a unique identifier σ that defines the cell volume V_σ_ and shape within the lattice [45]. To each cell identifier is also assigned a cell type τ(σ), in our case for instance a cancer stem cell or a differentiated cancer cell. In a CPM we can simulate cell proliferation and adhesion by representing these processes as transitions from energetically unfavorable states to energetically favorable ones. In this manner, for example, a compressed cell would seek to maintain its volume by creating a counterforce. The total energy of the system can be described by a simple Hamiltonian: 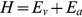
(1)with E_v_ the cell elastic energy and E_a_ the cell membrane contact energy. These variables correspond to the cost of a certain cell state in terms of energy.

### Volume elastic energy



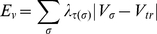
(2)Under no mechanical stress, the volume of a cell V_σ_ is equal to its *target volume* V_tr_, thus elastic energy E_v_ = 0. Under compression or stretch, the cell elastic energy increases as described by (2), where the cell elastic coefficient λ_τ(σ)_ depends only on the cell type τ(σ).

### Cell adhesion energy



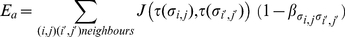
(3)The energy cost J(τ_1_,τ_2_) is assigned to each contact point of the cell membranes, and depends on the cell type. The term β in (3) avoids counting points belonging to the same cell. This method simulates cell membrane adhesion in a simple and elegant manner.

At each time step δt we evolve the system by randomly drawing a certain number of random local changes in a Monte Carlo fashion, those changes can be accepted or rejected as illustrated by the Metropolis algorithm below [46]:

1. Compute system energy *H*
2. Pick a random lattice site *(i,j)*
3. Set the content σ of *(i,j)* to that of its neighbor *(i',j')*, chosen at random
4. Calculate the energy difference *ΔH = H_new_ – H*
5. If *ΔH<0* accept new state because the total energy is lower
6. If *ΔH≥0* accept new state with probability 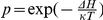

7. If cell is growing, increase target volume *V_tr_ = V_tr_+*δ*V*
8. If *V_σ_>2V_tr_* divide cell (*V_σ_* tries to grow to size *V_tr_* since it is a lower energy state)
9. Go to 1

This technique allows us to simulate efficiently and in an elegant way several crucial processes occurring in cancer. Our model proved to be very flexible and computationally fast and therefore suitable to perform statistical inference of biological parameters, as in the case of the colon crypt [47]. Here we propose an extension of such a model to simulate large cancer cell populations within a malignancy. The model simulates the expansion of malignant cells that originate from a single initial cancer cell that can proliferate by invading the surrounding tissue (not simulated) while accumulating DNA mutations according to any mutation scheme we need. Because cancers are accompanied by massive cell death we introduce in our model an apoptosis rate *a* that corresponds to the fraction of cells randomly selected for death every 24 h. This parameter allows us to investigate the influence of apoptosis in the dynamics of cancer epigenetic alterations in cancer.

On top of this tumor growth model, we implemented the CSC hierarchy by assuming two possible cell types in the system: cancer stem cells (CSC) and more differentiated cancer cells (DCC). At every division a CSC has probability δ to self-renew and therefore generate a new CSC, and probability 1-ψ to spin off a TAC. TACs can undergo a maximum of *G* divisions before becoming fully differentiated and irreversibly stopping division. By changing the parameter ψ we can modify the size of the CSC compartment, spanning tumors with a very small population of CSCs (ψ<1) to the classical model of malignancies where all cells in the tumor are tumorigenic and have stem-like features (ψ = 1). In this model no active cell migration is considered, however cells may diffuse due to low cell-to-cell adhesion and the distribution of mechanical forces within the tumor.

To implement cell fitness advantage, in relationship to a certain fitness landscape, we assign to each cell a specific phenotype in the range [−25,25] with the first cell starting with phenotype 0. To each phenotype *x* corresponds a fitness value *f(x)*, representing the growth rate of the cells possessing that phenotype. In the model this corresponds to the increase of target volume *δV* implemented in the step 7 illustrated above. In this way cells with a faster growing phenotype will reach the target volume faster and will consequently divide more often.

Although we have also developed a 3-dimensional version of the model, in this paper we make use of a 2-dimensional implementation of it. This represents a tumor as a sheet of cells and permits us to study the global evolutionary dynamics of a considerably larger cancer cell population by enhancing the computational feasibility. We believe that to investigate the dynamics we discuss, a two-dimensional model is a good and efficient solution, as for most tumor growth models [18], [32], [48], [49], [50], [51]. Moreover, agent-based models of tumor growth like the one we describe have proved to yield equivalent results in both two and three dimensions [18], [28]. In general, simulation times on a 2.5 GHz Xeon CPU were of ∼8 h for the classical model experiments and ∼20 h for the CSC model simulations assuming Ω = 2000×2000 with 16 points per cell.

### Measure of heterogeneity

To measure the level of clonal diversity in the tumor population we make use of the Shannon index of biodiversity [20] normalized to the interval [0,1]. The Shannon index is a measure of biodiversity of species within an ecosystem, in our case different methylation patterns within the tumor. A high value of the Shannon index indicates that all the species present in the ecosystem (the different methylation patterns in the malignancy) are present in equal numbers, a small Shannon index instead represents a scenario where one or few patterns are present in large numbers and virtually dominate the other patterns.

The Shannon index *H* is calculated as in equation (4),
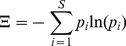
(4)where *p_i_* is the relative abundance of species *i* (a specific methylation pattern). For any given number of species *S* there is a maximum possible Shannon index of Ξ_max_ = *log S* that reflects the highest possible biodiversity in the system where all species are present in equal number. In our analysis we consider the normalized Shannon index Ξ' = Ξ/Ξ_max_ at any given point in time. According to this measure a value of 0 corresponds to the presence of a single dominant clone whereas a value of 1 represents a heterogeneous neoplasm where all the subclones are present in equal numbers.

## Supporting Information

Figure S1 Timescales and population size. The classical model (blue) displays faster growth rate (A) and larger amount of cell divisions per time (B) due to a fully tumorigenic population whereas the tumorigenic fraction in the CSC model (red) is limited to ∼0.2% of the total (C). Error bars represent SD with n = 16, see Table 1 for details on parameters used.
(0.63 MB EPS)

Figure S2 Heterogeneity of the cancer stem cell population. Increased heterogeneity for the CSC model is reported also with the standard Shannon index for the total population (A) as well as for the CSC compartment only (B). Error bars represent SD with *n = 16*.
(0.52 MB EPS)

Figure S3 Heterogeneity for different values of *G* and apoptosis rate. In both the classical (A) and the CSC model (B) the apoptosis rate modestly stimulates methylation pattern heterogeneity by inducing a larger number of cell divisions although in the CSC model this effect is significant (p = 0.009) whereas in the classical model it is not (p = 0.41). Different sizes of the TAC compartment have a non-trivial effect on heterogeneity (C) yet for the examined values only G = 7 significantly alters the results (p = 0.0002). Error bars represent SD with *n = 16*.
(0.64 MB EPS)

Figure S4 Robustness for variations of the mutation rate. The results are significantly robust with respect to changes in mutation rates although for lower mutation rates the differences are non significant due to the necessity to simulate much larger populations under very rare mutation events. Error bars represent SD with *n = 8*.
(0.46 MB EPS)

Figure S5 Evolution of non-neutral mutations at different growth stages. The evolutionary behavior of the two models is illustrated for growth points using the sinusoidal function *f_S_'(x)*. Here the mechanism of fitness landscape probing using genetic drift that characterizes the CSC model is evident from the change of distribution of the clones during growth. The volumes considered are 1k (A), 5k (B), 10K (C), 20k (D), 50k (E) and 100k (F). Error bars represent SD with *n = 8*.
(1.13 MB EPS)

Figure S6 The evolutionary dynamics of the CSC model are confirmed under constant population size and equal number of divisions. The same ability of the CSC model to overcome local maxima and achieve better fit in the long run while maintaining a pool of low-fitness clones is confirmed also when the population is maintained at constant size (25,000 cells) and compared to the classical model after 1.2M cell divisions. Error bars represent SD with *n = 8*.
(0.49 MB EPS)

Figure S7 Heterogeneity and average fitness of the two models. After treatment in both models the overall heterogeneity of the clones remains similar (A, CSC p = 0.46, classical p = 0.02). Yet the average fitness of the CSC model is dramatically increased in the regrown tumors (B, CSC p = 0.0023, classical p = 0.04). Error bars represent SD with *n = 12*.
(0.45 MB EPS)

Video S1 Simulated growth of a classical (flat) tumor.
(1.08 MB AVI)

Video S2 Simlated growth of a hierarchically organized tumor.
(3.36 MB AVI)

Video S3 Methylation patterns in a classical (flat) tumor.
(0.21 MB AVI)

Video S4 Methylation patterns in a hierarchically organized tumor.
(1.91 MB AVI)

## References

[1] Nowell PC (1976). The clonal evolution of tumor cell populations.. Science.

[2] Reya T, Morrison SJ, Clarke MF, Weissman IL (2001). Stem cells, cancer, and cancer stem cells.. Nature.

[3] Vermeulen L, Sprick MR, Kemper K, Stassi G, Medema JP (2008). Cancer stem cells–old concepts, new
insights.. Cell Death Differ.

[4] Singh SK, Hawkins C, Clarke ID, Squire JA, Bayani J (2004). Identification of human brain tumour initiating
cells.. Nature.

[5] Kern SE, Shibata D (2007). The fuzzy math of solid tumor stem cells: a
perspective.. Cancer Res.

[6] Tomasson MH (2009). Cancer stem cells: a guide for skeptics.. J Cell Biochem.

[7] Kelly PN, Dakic A, Adams JM, Nutt SL, Strasser A (2007). Tumor growth need not be driven by rare cancer stem
cells.. Science.

[8] Quintana E, Shackleton M, Sabel MS, Fullen DR, Johnson TM (2008). Efficient tumour formation by single human melanoma
cells.. Nature.

[9] Hill RP (2006). Identifying cancer stem cells in solid tumors: case not
proven.. Cancer Res.

[10] Adams JM, Strasser A (2008). Is tumor growth sustained by rare cancer stem cells or dominant
clones?. Cancer Res.

[11] Shackleton M, Quintana E, Fearon ER, Morrison SJ (2009). Heterogeneity in cancer: cancer stem cells versus clonal
evolution.. Cell.

[12] Siegmund KD, Marjoram P, Shibata D (2008). Modeling DNA methylation in a population of cancer
cells.. Stat Appl Genet Mol Biol.

[13] Woo YJ, Siegmund KD, Tavaré S, Shibata D (2009). Older individuals appear to acquire mitotically older colorectal
cancers.. J Pathol.

[14] Siegmund KD, Marjoram P, Woo YJ, Tavaré S, Shibata D (2009). Inferring clonal expansion and cancer stem cell dynamics from DNA
methylation patterns in colorectal cancers.. Proc Natl Acad Sci U S A.

[15] Yatabe Y, Tavaré S, Shibata D (2001). Investigating stem cells in human colon by using methylation
patterns.. Proc Natl Acad Sci U S A.

[16] Kunkel TA, Bebenek K (2000). DNA replication fidelity.. Annu Rev Biochem.

[17] Shibata D (2009). Inferring human stem cell behaviour from epigenetic
drift.. J Pathol.

[18] Sottoriva A, Verhoeff JJ, Borovski T, McWeeney SK, Naumov L (2010). Cancer stem cell tumor model reveals invasive morphology and
increased phenotypical heterogeneity.. Cancer Res.

[19] Enderling H, Hlatky L, Hahnfeldt P (2009). Migration rules: tumours are conglomerates of
self-metastases.. Br J Cancer.

[20] Shannon CE (1948). A mathematical theory of communication.. Bell Systems Technical Journal.

[21] Weissman DB, Desai MM, Fisher DS, Feldman MW (2009). The rate at which asexual populations cross fitness
valleys.. Theoretical Population Biology.

[22] Sensi M, Nicolini G, Petti C, Bersani I, Lozupone F (2006). Mutually exclusive NRASQ61R and BRAFV600E mutations at the
single-cell level in the same human melanoma.. Oncogene.

[23] Weinreich DM, Chao L (2005). Rapid evolutionary escape by large populations from local fitness
peaks is likely in nature.. Evolution.

[24] Trumpp A (2006). c-Myc and activated Ras during skin tumorigenesis: cooperation at
the cancer stem cell level?. Ernst Schering Found Symp Proc.

[25] Draghi JA, Parsons TL, Wagner GP, Plotkin JB (2010). Mutational robustness can facilitate adaptation.. Nature.

[26] Roose T, Chapman SJ, Maini PK (2007). Mathematical models of avascular tumor growth.. Siam Review.

[27] Dormann S, Deutsch A (2002). Modeling of self-organized avascular tumor growth with a hybrid
cellular automaton.. In Silico Biol.

[28] Anderson AR, Weaver AM, Cummings PT, Quaranta V (2006). Tumor morphology and phenotypic evolution driven by selective
pressure from the microenvironment.. Cell.

[29] Sottoriva A, Sloot PM, Medema JP, Vermeulen L (2010). Exploring cancer stem cell niche directed tumor
growth.. Cell Cycle.

[30] Wu FY (1982). The Potts Model.. Rev Mod Phys.

[31] Tripodi S, Ballet P, Rodin V Computational Energetic Model of Morphogenesis Based on
Multi-agent Cellular Potts Model.. Adv Exp Med Biol.

[32] Jiang Y, Pjesivac-Grbovic J, Cantrell C, Freyer JP (2005). A multiscale model for avascular tumor growth.. Biophys J.

[33] Merlo LM, Pepper JW, Reid BJ, Maley CC (2006). Cancer as an evolutionary and ecological process.. Nat Rev Cancer.

[34] Aubele M, Mattis A, Zitzelsberger H, Walch A, Kremer M (1999). Intratumoral heterogeneity in breast carcinoma revealed by
laser-microdissection and comparative genomic hybridization.. Cancer Genet Cytogenet.

[35] Navin N, Krasnitz A, Rodgers L, Cook K, Meth J (2010). Inferring tumor progression from genomic
heterogeneity.. Genome Res.

[36] Park SY, Gonen M, Kim HJ, Michor F, Polyak K (2010). Cellular and genetic diversity in the progression of in situ
human breast carcinomas to an invasive phenotype.. J Clin Invest.

[37] Calabrese C, Poppleton H, Kocak M, Hogg TL, Fuller C (2007). A perivascular niche for brain tumor stem cells.. Cancer Cell.

[38] Vermeulen L, De Sousa EMF, van der Heijden M, Cameron K, de Jong JH (2010). Wnt activity defines colon cancer stem cells and is regulated by
the microenvironment.. Nat Cell Biol.

[39] Maley CC, Galipeau PC, Finley JC, Wongsurawat VJ, Li X (2006). Genetic clonal diversity predicts progression to esophageal
adenocarcinoma.. Nat Genet.

[40] Gokhale CS, Iwasa Y, Nowak MA, Traulsen A (2009). The pace of evolution across fitness valleys.. J Theor Biol.

[41] Huff CA, Matsui W, Smith BD, Jones RJ (2006). The paradox of response and survival in cancer
therapeutics.. Blood.

[42] Spiegl-Kreinecker S, Pirker C, Marosi C, Buchroithner J, Pichler J (2007). Dynamics of chemosensitivity and chromosomal instability in
recurrent glioblastoma.. Br J Cancer.

[43] El Sharouni SY, Kal HB, Battermann JJ (2003). Accelerated regrowth of non-small-cell lung tumours after
induction chemotherapy.. Br J Cancer.

[44] Lee J, Kotliarova S, Kotliarov Y, Li A, Su Q (2006). Tumor stem cells derived from glioblastomas cultured in bFGF and
EGF more closely mirror the phenotype and genotype of primary tumors than do
serum-cultured cell lines.. Cancer Cell.

[45] Glazier JA, Graner F (1993). Simulation of the differential adhesion driven rearrangement of
biological cells.. Phys Rev E Stat Phys Plasmas Fluids Relat Interdiscip Topics.

[46] Beichl I, Sullivan F (2000). The metropolis algorithm.. Computing in Science & Engineering.

[47] Sottoriva A, Tavaré S (2010). Integrating approximate bayesian computation with complex
agent-based models for cancer research..

[48] Anderson AR, Chaplain AJ, Newman EL, Steele RJ, Thompson AM (2000). Mathematical modeling of tumor invasion and
metastasis.. jounal of theoretical medicine.

[49] Anderson AR (2005). A hybrid mathematical model of solid tumour invasion: the
importance of cell adhesion.. Math Med Biol.

[50] Bearer EL, Lowengrub JS, Frieboes HB, Chuang YL, Jin F (2009). Multiparameter computational modeling of tumor
invasion.. Cancer Res.

[51] Macklin P, McDougall S, Anderson AR, Chaplain MA, Cristini V (2009). Multiscale modelling and nonlinear simulation of vascular tumour
growth.. J Math Biol.

[52] Tomita K, Plager JE (1979). In vivo cell cycle synchronization of the murine sarcoma 180
tumor following alternating periods of hydroxyurea blockade and
release.. Cancer Res.

[53] Rubenstein BM, Kaufman LJ (2008). The role of extracellular matrix in glioma invasion: a cellular
Potts model approach.. Biophys J.

